# Computer Vision-Based Approach for Quantifying Occupational Therapists' Qualitative Evaluations of Postural Control

**DOI:** 10.1155/2020/8542191

**Published:** 2020-04-27

**Authors:** Hiromichi Hagihara, Naoto Ienaga, Daiki Enomoto, Shuhei Takahata, Hiroyuki Ishihara, Haruka Noda, Koji Tsuda, Kei Terayama

**Affiliations:** ^1^Graduate School of Human and Environmental Studies, Kyoto University, Kyoto, Japan; ^2^Research Fellow of Japan Society for the Promotion of Science, Tokyo, Japan; ^3^Graduate School of Science and Technology, Keio University, Yokohama, Japan; ^4^LITALICO Inc., Tokyo, Japan; ^5^Department of Rehabilitation, Hakuho College, Nara, Japan; ^6^Nippon Telegraph and Telephone, Kanagawa, Japan; ^7^Graduate School of Biomedical Sciences, Nagasaki University, Nagasaki, Japan; ^8^Graduate School of Frontier Science, University of Tokyo, Tokyo, Japan; ^9^RIKEN Center for Advanced Intelligence Project, Tokyo, Japan; ^10^Medical Sciences Innovation Hub Program, RIKEN Cluster for Science, Technology and Innovation Hub, Kanagawa, Japan; ^11^Graduate School of Medicine, Kyoto University, Kyoto, Japan

## Abstract

This study aimed to leverage computer vision (CV) technology to develop a technique for quantifying postural control. A conventional quantitative index, occupational therapists' qualitative clinical evaluations, and CV-based quantitative indices using an image analysis algorithm were applied to evaluate the postural control of 34 typically developed preschoolers. The effectiveness of the CV-based indices was investigated relative to current methods to explore the clinical applicability of the proposed method. The capacity of the CV-based indices to reflect therapists' qualitative evaluations was confirmed. Furthermore, compared to the conventional quantitative index, the CV-based indices provided more detailed quantitative information with lower costs. CV-based evaluations enable therapists to quantify details of motor performance that are currently observed qualitatively. The development of such precise quantification methods will improve the science and practice of occupational therapy and allow therapists to perform to their full potential.

## 1. Introduction

The aim of occupational therapy is to promote people's participation in daily activities [1]. This therapy is offered to a wide range of clients, from children to the elderly. Occupational therapists conduct holistic assessments of clients' current body functions, activities, and participation in daily lives and draw mutual relationships among these aspects. In the field of paediatric or school occupational therapy, in particular, the relationships between body functions such as postural control and everyday activities are very important because body functions form the basis for the occupational performance of children with regard to skills such as self-care, communication, and subject learning [2]. In fact, impaired postural control has been widely reported in people diagnosed with developmental disorders, such as autism spectrum disorder [3–5], attention-deficit/hyperactivity disorder [6, 7], and developmental coordination disorder [8, 9]. Therefore, it is essential to devise evaluation indices of body functions that can enable therapists to grasp these difficulties comprehensively. Such indices also help to establish evidence of the usefulness of occupational therapy in developing outcome measures, which are sensitive to occupational therapy interventions [10].

However, current evaluation indices have serious limitations, especially the quantitative ones. First, the granularity of conventional quantification methods is coarse. In the clinical practice of paediatric occupational therapy, oft-used assessments such as the Movement Assessment Battery for Children 2 [11], the Bruininks-Oseretsky Test of Motor Proficiency 2 [12], or the Japanese Playful Assessment for Neuropsychological Abilities [13] generate quantitative outcomes that are simply the number of task successes, duration time, or speed. Although these indices can be useful, they are not sufficient to detail critical motor performances such as balance, antigravity posture, or compensatory movement, all of which therapists instead observe qualitatively. Second, the detailed qualitative evaluations that the occupational therapist would normally perform in the above assessments are often excluded from the indices themselves because such evaluations are considered to be difficult to quantify. Although certain evaluation methods can quantify such qualitative viewpoints using specialized equipment (e.g., body sway meters), these methods are typically quite expensive and have poor clinical availability [14]. Recently, some solutions have been proposed such as evaluations using consumer electronics devices [15, 16]. However, occupational therapists still seem to adopt practical classification methods based on certain criteria for their clinical convenience instead of quantifying the quality of motor performances. Third, current motor skill evaluation procedures require much time and impose physical and mental burdens on patients, resulting in increased exhaustion and reduced motivation [17]. In particular, postural control basically needs the ability to maintain postural balance as well as the ability to maintain positions of body parts against the force of gravity to keep posture stable. Therapists have utilized different tasks to evaluate these abilities independently (e.g., one-foot standing for static postural balance and prone extension for antigravity posture, respectively) [18, 19]. The development of techniques that enables therapists to observe these abilities simultaneously in one task can reduce time costs and the burdens of patients.

In this research, we investigate the application of computer vision (CV) technology to resolve these problems. Recent advances in deep learning technology have made significant progress in the field of monocular human posture estimation [20–23]. These methods estimate the 2D or 3D positions of human key points (e.g., joints) from only RGB images or movies. CV studies capitalizing on such pose estimation methods enabled researchers to easily quantify human postures or body movements [24–26]. In the past several years, CV techniques have been applied to medical fields which include, for example, diagnosis of autism spectrum disorder [27, 28] or detection of gait abnormalities [29]. Although detailed quantitative analyses of postures and body movements have conventionally required special equipment such as motion captures or force plates, these technological innovations have the potential to decrease their expense and ease their implementation by therapists in the clinical setting.

The purpose of this research was to develop a technique for quantifying postural control by using a pose estimation algorithm called OpenPose [20] and to explore the proposed technique's clinical applicability by comparing it to individual occupational therapists' qualitative clinical evaluations. As the first step toward this goal, we conducted a simple postural control evaluation, a static posture maintenance task, for typically developing children.

This research contributes to the occupational therapy field first by demonstrating the possibility of more accurate and detailed quantitative evaluations, and second by simplifying conventional evaluation tasks with the elimination of special equipment and reduced time investments, imposing less burden on clients. Furthermore, this research may accelerate collaborations between occupational therapy and computer vision fields.

## 2. Materials and Methods

### 2.1. Design

**Figure 1:**
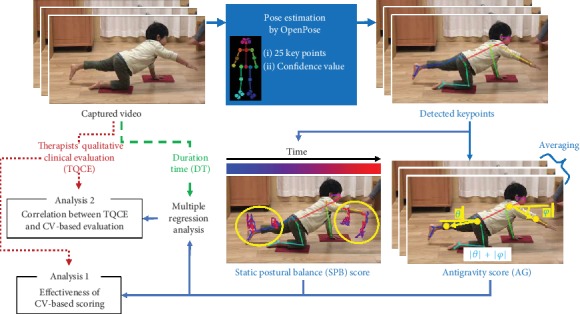
Research flowchart.

Typically developed children performed a developmental task standardised in Japan to evaluate postural stability, and three types of indices were calculated: (1) a conventional quantitative index measuring duration time (DT), (2) therapists' qualitative clinical evaluations (TQCE) using the 7-point Likert scale, and (3) CV-based quantitative indices based on image analysis algorithms for static postural balance (SPB) and antigravity (AG). We then investigated how closely the CV-based scores reflected the TQCE (analysis 1), and how effective the CV-based model was compared to the DT-based model (analysis 2; Figure 1). As the collaborative and interdisciplinary research, both occupational therapists and CV engineers participated in every step of the research flow in order to avoid mutual lack of understanding due to the explicit division of work.

### 2.2. Participants

Thirty-four preschoolers (14 girls; mean age = 4.7; SD = 1.0; range = 3–6 years old) from two nursery schools in Japan participated in this research. An additional five children also participated but were excluded from the final sample because of their difficulty maintaining the task posture without assistance. All participants in this research were considered to be developing typically. Each participant's parents provided written informed consent, and this research was approved by the Research Ethics Committee of Hakuho College (18012).

### 2.3. Instrumentation

The Japanese Playful Assessment for Neuropsychological Abilities (JPAN) is a developmental assessment battery for evaluating children's sensory integration abilities [13, 30, 31], which was developed in Japan based on previous tests developed in the United States, such as the Sensory Integration and Praxis Tests [32], and standardised from a large sample (489 Japanese children of 4–10 years old). The JPAN consists of 32 subtests (six for equilibrium and antigravity posture, seven for somatosensory, 15 for praxis, and four for eye-hand coordination and visual perception), and these have been confirmed for high interrater reliability (intraclass correlations in the range of 0.81–1.00 for each subtest) and internal consistency (Cronbach's alpha of 0.75 for all subtests) [33].

Among JPAN subtests, we selected the One Arm and One Leg Balance as the experimental task for this research, in the domain of equilibrium and antigravity posture. This task required a participant to maintain what is called “bird dog posture” for up to 60 s on each side, in which he/she lifted the arm on one side and the leg on the other side from a four-point crawling posture (see Figure 1). This task required an unfamiliar balance [34] and was therefore considered to remove the influence of participants' splinter skills to some extent. Despite the need for multiple functions to maintain this posture, including static postural balance and antigravity, the task has been quantitatively assessed only by DT, which was the primary reason for selecting this task.

### 2.4. Task Procedure

Each participant worked on the task individually in a vacant room in a nursery school, performing the task twice (once on each side) according to the JPAN manual (for up to 60 s on each side). The examiner cheered participants up to encourage them to maintain the bird dog posture as long as they could (e.g., “Lift your arm!”). Task performances were videotaped from the side and at approximately the same height on each participant's trunk, so that video frames included each participant's whole body on the sagittal plane.

### 2.5. Applied Indices

Three types of indices were calculated to explore which indices most appropriately reflected therapists' qualitative clinical evaluations.

#### 2.5.1. DT

The DT for which each participant maintained the posture was measured by detecting the frames in which participants fully extended their arms and legs, and those in which any part of these body parts was attached to the floor. If 60 s elapsed without putting any part of these body parts on the floor, the task was aborted, and the duration time was recorded as 60 s. This index is a conventional quantitative outcome used in the JPAN.

#### 2.5.2. TQCE

Three paediatric occupational therapists (Hagihara, Noda, and Takahata, authors of this article; their clinical experiences were approximately 4, 2, and 11, respectively) evaluated the quality of the task performance based on the clinical viewpoint of each therapist following the 7-point Likert scale (where a score of 7 indicates the most superior performance). These therapists watched and scored the recorded videos individually so that they did not know other therapists' scoring. In addition, they watched the raw recorded videos that had not been analyzed by OpenPose yet. Hence, they did not know the outcomes of CV-based quantitative indices for each participant at all when evaluating. A high interrater reliability was confirmed (ICC (2, 1) = 0.892). The three therapists' evaluations were averaged to determine the TQCE index.

#### 2.5.3. CV-Based Approach

First, three CV engineers (Ienaga, Terayama, and Ishihara, authors of this article) conducted the key point detection of body parts with OpenPose version 1.4.0. OpenPose is an open-source library for real-time multiperson key point detection of a body, face, and hands using a convolutional neural network [20]. Using a method called Part Affinity Fields (PAFs), OpenPose can take into account the positional relationship of each key point, that is, the human skeletal structure. This algorithm contributes to improving the accurate estimation of key-point positions. OpenPose can estimate the 2D positions of 25 body key points, which roughly correspond to joints (e.g., key point 6 is left elbow; see Figure 1). The confidence value of each key point for each frame is automatically calculated, ranging from 0 to 1 (a higher value indicates higher confidence), so that the degree of precision of key-point estimation can be comprehensible. For example, in Figure 1, all the key points used for subsequent analysis were detected with high confidence values (>0.5).

OpenPose can detect the key points more accurately when the head of a person is above and the legs below due to the effect of the training data. The input video was rotated 90° to turn the participants' heads upward since the participants kept the bird dog posture as shown in Figure 1. The key points were then rotated 90° back after being detected by OpenPose. To eliminate the detection noise, key points whose confidence values were less than 0.5 were removed in each frame and interpolated linearly.

Because occupational therapists evaluate SPB and AG in addition to duration time, we defined these items as shown below.

#### 2.5.4. Static Postural Balance (SPB)

We first calculate the moving distance *D*′.

(1) $$\begin{array}{c} D=\frac{1}{n-1}\overset{n-1}{\underset{i=1}{\sum }}\frac{d_{\text{i}-1}^{\text{i}}}{bl}, \end{array}$$

(2) $$\begin{array}{c} D^{\prime }=\left(D_{\text{w}}+D_{\text{e}}+D_{\text{a}}+D_{\text{k}}\right)/4. \end{array}$$

*D* is the moving distance of a key point averaged in the frame direction (*n* represents the number of frames of the video). The moving distance *d*_i−1_^i^ between two consecutive frames *i*, *i* − 1 is divided by the body length *bl*. The body length is the average value of the distance between two key points corresponding to the neck and hip in all frames of the video. The reason for dividing the moving distance by the body length is to reduce the influences of the differences in the heights of the subjects and in the distances between the camera and the subjects. There are actually four *D*s: for the wrist key point *D*_*w*_ and the elbow key point *D*_*e*_ of the extended arm, and the ankle key point *D*_*a*_ and the knee key point *D*_*k*_ of the extended leg. SPB is the standardised value (*z*-score) obtained as follows:

(3) $$\begin{array}{c} SPB=\frac{\log\left(1/D^{\prime }\right)-\mu }{\sigma }, \end{array}$$

where *μ* and *σ* indicate the average and standard deviation of log(1/*D*′), respectively. The average and standard deviation of SPB are 0 and 1, respectively, because SPB is standardised to eliminate the influence of numerical magnitude for multiple regression analysis with other indices. High SPB indicates high static postural stability.

#### 2.5.5. Antigravity Posture (AG)

AG was calculated as

(4) $$\begin{array}{c} a=-\frac{1}{n}\overset{n-1}{\underset{i=0}{\sum }}\left(\left|\varphi \right|+\left|\theta \right|\right), \end{array}$$

where |*φ*| and |*θ*| are the absolute angles between the floor and the extended arm and leg, respectively, as shown in Figure 1. Here, “arm” refers to the vector connecting the neck position to the average position of the wrist and elbow, and “leg” refers to the vector connecting the hip position to the average position of the ankle and knee. Because these are absolute values, the angle increases whether the limb is raised or lowered. *a* is the negative of the sum of the two angles averaged over the frame direction, and AG is the standardised value (*z*-score) of *a*. High AG indicates high antigravity posture.

### 2.6. Data Analysis

First, we investigated how closely the CV-based scores (SPB and AG) reflected TQCE using Spearman's rank correlation coefficient (analysis 1). Next, we compared the effectiveness of the CV-based model to that of the DT-based model (analysis 2). SPB, AG, and DT together or DT alone were used as the explanatory variables, and regression analyses were performed using TQCE as the objective variable, with subsequent correlation analysis between the predicted value of the regression analysis and TQCE, assuming that the test time was unchanged.

## 3. Results and Discussion

### 3.1. Degree to which CV-Based Scores Reflected TQCE

**Figure 2:**
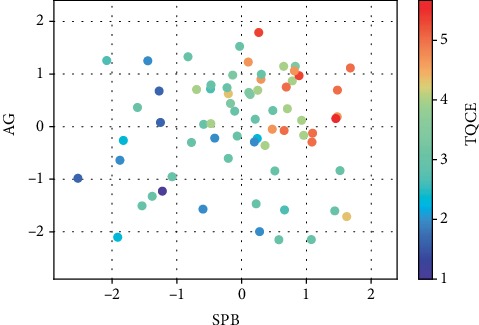
Scatter plot of SPB and AG with colour bar of TQCE.

Based on the analyses using Spearman's rank correlation coefficient, SPB showed a significant positive correlation with TQCE (*rs* = 0.654, *p* < 0.001). AG also showed a significant, though weak, positive correlation with TQCE (*rs* = 0.382, *p* = 0.0013). TQCE tended to be higher when both of these CV-based scores were high (Figure 2).

### 3.2. Multiple Regression Model Reflecting TQCE

To compare the evaluations using only the conventional quantitative index (DT) with the evaluations using all three indices (DT and the CV-based scores), we set TQCE as the objective variable and calculated the model using DT as the explanatory variable in a single regression analysis, in comparison with the model using SPB, AG, and DT as the explanatory variables in a multiple regression analysis. DT alone had a significant positive correlation with TQCE (*rs* = 0.886, *p* < 0.001). However, the value calculated by multiple regression analysis, which included the CV-based scores, showed a stronger significant positive correlation with TQCE (*rs* = 0.914, *p* < 0.001).

**Figure 3:**
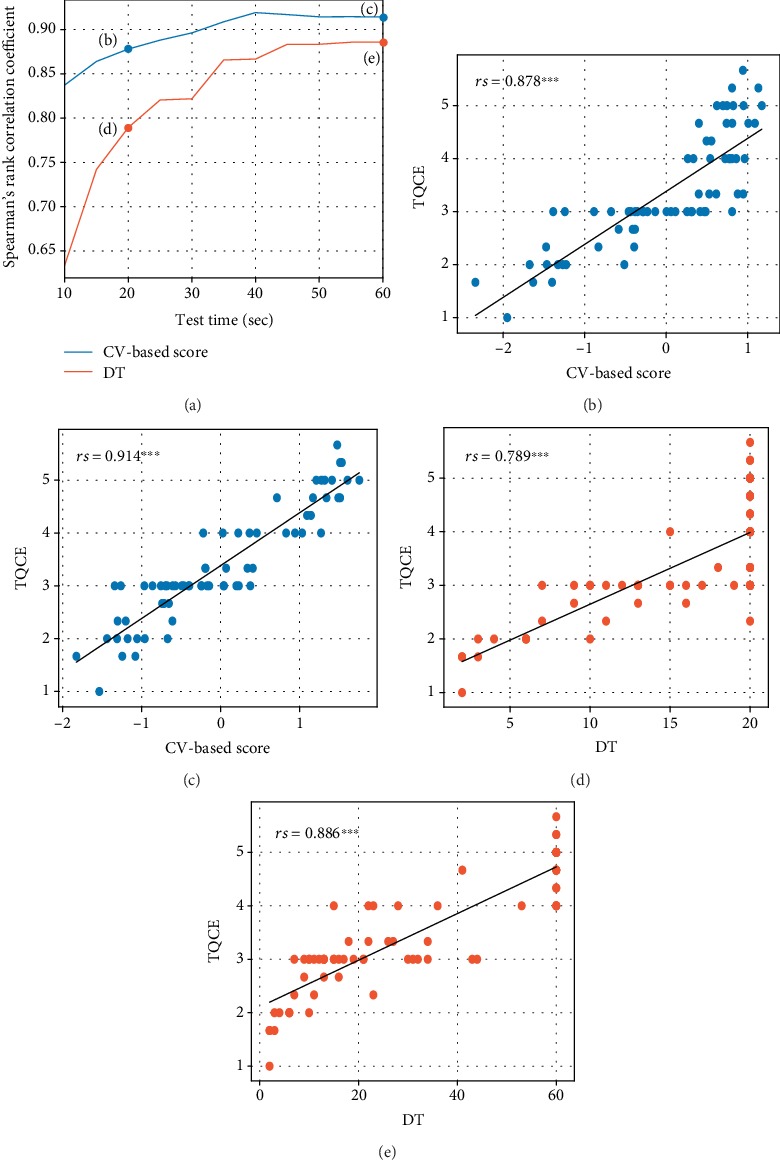
Comparison of the CV- and DT-based models. (a) Correlation coefficient between TQCE and CV- (blue) or DT-based (orange) models for every 10 s of test time. (b) Scatter plot of CV-based scores and TQCE at the test time of 20 s. (c) Scatter plot of CV-based scores and TQCE at the test time of 60 s. (d) Scatter plot of DT-based scores and TQCE at the test time of 20 s. (e) Scatter plot of DT-based scores and TQCE at the test time of 60 s. ^∗∗∗^*p* < 0.001.

For test times shorter than 60 s, the difference between the CV- and DT-based scores was even more pronounced (Figure 3(a)). When the test time was reduced to 20 s, the CV-based score showed a higher positive correlation (*rs* = 0.878, *p* < 0.001; see Figures 3(b) and 3(c)) than DT alone (*rs* = 0.789, *p* < 0.001; see Figures 3(d) and 3(e)). In all 68 trials (34 participants × two sides), the number of trials for which DT was equal or more than 60 s was 18 (26%), and the number for which DT was equal or more than 20 s was 37 (54%). The results of each regression analysis are shown in Table 1.

### 3.3. Effectiveness of CV-Based Indices

The results of this research revealed the following two points. First, both SPB and AG had a significant positive correlation with TQCE, and TQCE tended to be higher when both of these CV-based scores were high, indicating that these indices quantified TQCE well. Second, the multiple regression analysis and subsequent correlation analysis showed that the CV-based model reflected the TQCE to a greater degree than the conventional DT-based model, indicating that evaluations using CV-based scores provide more detailed quantitative information. These results showed the possibility of overcoming the severe problems with current quantitative evaluation indices, which are useful for screening but are not sufficient for verifying clinical effects or depicting details of motor performances including postural control. Although a certain proportion of children with developmental disorders have difficulties with postural control [3–9], they often demonstrate the maximum performance in terms of current quantitative scores, resulting in a ceiling effect [17], and their difficulties with postural control can only be identified by therapists' clinical observations. By using CV-based scores, therapists can quantitatively evaluate their postural control even when they achieve maximum DT. Although the correlation coefficient between AG and TQCE was significant but low, this result may have occurred because therapists evaluated AG posture only after confirming the achievement of initial evaluation items, such as durability or static postural balance.

The developed CV-based scores, SPB and AG, will contribute not only to identifying essential factors of postural control that have negative effects on children's everyday activities, but also to establishing more valid evidence for the efficacy of occupational therapy. Body functions such as postural control are the basis for children's occupational performance on skills such as self-care, communication, and subject learning [2]. Furthermore, children with developmental disorders, especially developmental coordination disorder, often suffer from psychological problems, including lower self-esteem [35, 36], anxiety, and depression [37, 38]. The qualitative improvement of postural control might thus contribute to improving their activities and participation, and such improvements might be grasped by CV-based scores because these detailed quantitative indices reflect the qualitative aspects of postural control. These scores will also meet the need for developing functional outcome measures, which are sensitive to occupational therapy interventions [10]. Studies on the postural control of people with developmental disorders have focused on a specific posture only, such as standing on one leg (e.g., [39]) or on both legs (e.g., [40, 41]), the results of which cannot be widely applied to various postures. In contrast, the CV-based approach will broaden opportunities to advance detailed and quantitative evaluations of the qualitative aspects of a broad range of postures important for early postural development and activities in daily living.

### 3.4. Applicability to the Development of Lower Cost and more Detailed Assessments

The degree to which the CV-based model reflected TQCE was hardly reduced even when the test time was shortened. Furthermore, the precision with which the models reflected TQCE were almost equal between the DT-based model at the test time of 60 s and the CV-based model at the test time of 20 s. This suggests the possibility of making the current assessment task procedure easier and simpler to reduce the time cost of testing and the physical and psychological burden on clients. In fact, the high cost and burden of conventional standardised assessments for children with poor motor skills are regarded as a clinical problem because longer test times cause fatigue and loss of motivation [17]. The CV-based approach shortens the test time and also yields more detailed quantitative information that is useful for both scientific research and clinical practice.

The experimental task, “One Arm and One Leg Balance,” used in this research simultaneously evaluates multiple aspects of postural control including at least SPB, AG, and DT. Although developing several tasks to evaluate each aspect separately may be desirable in particularly complex cases, this increases the cost and physical and psychological burdens on patients and thus has poor clinical applicability. The research presented herein demonstrates that the proposed indices resolve and quantify multiple aspects of postural control for multiple important aspects simultaneously, offering substantial reductions in the cost of the evaluation without compromising the amount of information.

### 3.5. Limitations and Future Work

This research has several limitations because we conducted a simple task on typically developing children so that the clinical applicability of CV-based quantification and analysis could be easily explored. A larger and randomized sample of children with and without developmental disorders must be beneficial to confirm the validity of this CV-based approach. Furthermore, the relationship between the seriousness of motor skill impairment or its developmental change and CV-based indices should be investigated for diagnostic use. Whether the indices for postural control developed in this research can be applied to other tasks or postures must also be confirmed. Application software that facilitates therapists' quantitative evaluations or verifications of the effectiveness of interventions would be especially important clinically. Despite these limitations, this research advances the fusion of occupational science and practice by bringing together the fields of occupational therapy and CV. In future works, we are planning to focus on the clinical usability of the CV-based approach proposed in this research (e.g., user interface). Furthermore, we intend to develop a versatile quantification approach that can be used not only in standardised assessments but also in actual occupational activities and is applicable to various disorders. The development of more precise quantification methods provides a basis for considering more effective interventions and contributes to realizing occupational therapists' full potential.

## 4. Conclusions

This study confirmed the close match between the results of CV-based indices (SPB and AG) and TQCE. Furthermore, compared with assessments using only the conventional quantitative index (DT), evaluations that included CV-based indices provided more detailed quantitative information with lower time costs. This research showed the possibility of overcoming the problems associated with current quantitative evaluations: that they are insufficient for verifying clinical effects or detailing motor performances, which therapists must observe qualitatively. The results of this study have the following implications for occupational therapy practice:

- Application of CV-based indices provides detailed quantitative and shareable information useful for not only screening assessments but also for verifying interventions more precisely
- CV-based approaches can reduce the time cost and the physical and psychological burdens on clients imposed by the performance of the assessment task
- Leveraging CV technology enables the direct quantitative evaluation of motor performance during actual occupational activities of daily living

This research also showed the possibility of making conventional evaluation tasks easier and simpler without special equipment so that therapists can reduce time costs and the physical and psychological burdens on clients. This integration of occupational therapy and CV advances the science and practice of occupational therapy so that therapists can perform to their full potential.

## Figures and Tables

**Table 1:** Regression analysis results.

	Model using SPB, AG, and DT				Model using only DT			
	Test time: 20 s		Test time: 60 s		Test time: 20 s		Test time: 60 s	
Variables	*β*	(*SE*)	*β*	(*SE*)	*β*	(*SE*)	*β*	(*SE*)
SPB	0.35	(0.083)^∗∗∗^	0.25	(0.059)^∗∗∗^				
AG	0.25	(0.070)^∗∗∗^	0.22	(0.050)^∗∗∗^				
DT	0.50	(0.068)^∗∗∗^	0.69	(0.060)^∗∗∗^	0.76	(0.081)^∗∗∗^	0.88	(0.059)^∗∗∗^
Adjusted *R*^2^	0.69^∗∗∗^		0.84^∗∗∗^		0.57^∗∗∗^		0.77^∗∗∗^	

^∗∗∗^ *p* < 0.001; therapists' qualitative clinical evaluation (TQCE) was set as the objective variable. SPB: static postural stability; AG: antigravity posture; DT: duration time; SE: standard error.

## Data Availability

The data used to support the findings of this study are Excel files, which consist of DT, CV-based indices (SPB and AG), and TQCE of each participant. Please contact the authors of the article to address the data.
